# Examining How Clinicians Make Judgements About Clinical ‘Caseness’ in Routine Practice: A Qualitative Study of Decision-Making in Acute Psychiatric Scenarios

**DOI:** 10.1192/bjo.2026.11194

**Published:** 2026-06-30

**Authors:** Anusha Akella, Rachel Sevillano, Rajan Nathan

**Affiliations:** 1 Cheshire and Wirral Partnership, NHSFT, Chester, United Kingdom; 2 University of Chester, Chester, United Kingdom; 3 Liverpool John Moore University, Liverpool, United Kingdom

## Abstract

**Aims:**

The effective implementation of evidence-based interventions is heavily dependent on matching interventions correctly to patients’ clinical needs, which, in turn, relies on the way the patients’ clinical needs are understood. Although previous research indicates a high degree of variability in assessment approaches, it remains unclear how clinicians determine clinical ‘caseness’ in routine practice. The aim of this study was to examine how clinicians make judgements about the nature of patients’ presentations when making care-related decisions in acute scenarios.

**Methods:**

Semi-structured focus groups were undertaken with clinicians whose primary roles included assessments and decision-making in acute psychiatric scenarios. Participants were presented with a real-life vignette of a complex yet common presentation and asked to discuss their approaches to the assessment, conceptualisation and understanding a range of patient experiences illustrated in the vignette (with a particular focus on how the participant understands these experiences from perspective of phenomenology, psychopathology, causation and diagnosis). The interviews were recorded, transcribed and anonymised and the transcripts were subject to thematic analysis.

**Results:**

The results reported were based on an analysis of data from 4 focus groups involving a total of n=21 participants.

1. The thematic analysis of the way clinicians reached judgements about clinical ‘caseness’ identified four main themes:

2. Specific terminology to convey the relative clinical significance of patient experiences (e.g. ‘intrusive thoughts’ versus ‘voices’ or ‘hallucinations’).

3. Informal rules to reach a judgement about the clinical significance of signs and symptoms (e.g. rules based on the patient’s demeanour to determine whether or not to accept a patient’s self-report at face value).

4. Judgements about the typicality of the self-report.

5. Judgements based on causal assumptions about patients’ presentations (e.g. ‘drug-induced’ or ‘personality disorder voices’).

**Conclusion:**

The findings of this study indicate that in routine mental health practice clinicians reach judgements about the clinical significance of patients’ presentation by using a range of assumptions that are outwith conventional guidance for assessing mental health morbidity. If these findings are supported by the results of other studies, then consideration should be given to (i) developing and implementing agreed approaches to determining clinical caseness in routine practice and (ii) ensuring that implementation research includes an evaluation of the impact of caseness-related judgements on the effectiveness of interventions in routine care.

